# Structural characterization of EasH (*Aspergillus japonicus*) – an oxidase involved in cycloclavine biosynthesis†
†Electronic supplementary information (ESI) available. See DOI: 10.1039/c6cc08438a



**DOI:** 10.1039/c6cc08438a

**Published:** 2016-11-22

**Authors:** Dorota Jakubczyk, Lorenzo Caputi, Clare E. M. Stevenson, David M. Lawson, Sarah E. O'Connor

**Affiliations:** a Department of Biological Chemistry , John Innes Centre , Norwich Research Park , Norwich NR4 7UH , UK . Email: Sarah.oconnor@jic.ac.uk

## Abstract

Aj_EasH is a non-heme iron- and α-keto-glutarate-dependent oxidase that is responsible for an unusual cyclopropyl ring formation in the biosynthesis of the fungal ergot alkaloid cycloclavine. The three dimensional structure of Aj_EasH (2.2 Å resolution) reported here provides insight into the mechanism of this unusual and complex reaction.

## Abstract 2

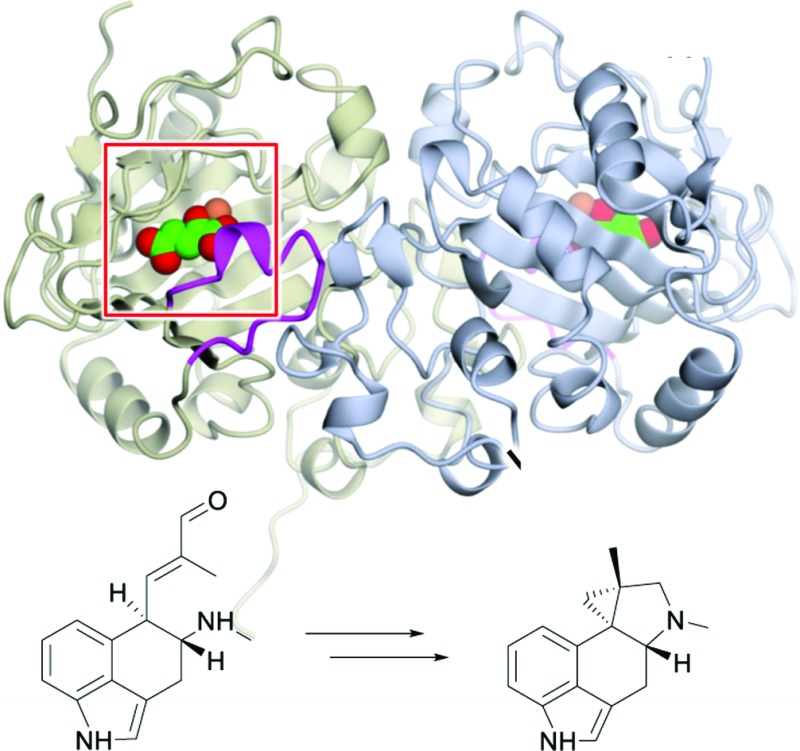
Aj_EasH is a non-heme iron- and α-keto-glutarate-dependent oxidase that is responsible for an unusual cyclopropyl ring formation in the biosynthesis of the fungal ergot alkaloid cycloclavine.

EasH from *Aspergillus japonicus* (Aj_EasH) is a recently discovered enzyme involved in the biosynthesis of the natural product cycloclavine **4**.^1,2^ This compound is a member of the ergot alkaloids, a fungal-derived class of natural products with a wide range of biological activities and pharmaceutical applications.^3–9^ Aj_EasH is a non-heme iron, α-keto-glutarate (aKG) dependent oxidase that works together with a flavoenzyme (EasA) and an NADPH-dependent oxidoreductase (EasG).^2,10–12^ In concert, these three enzymes catalyze the conversion of chanoclavine-I aldehyde **1** to cycloclavine **4** (Fig. 1).

**Fig. 1:**
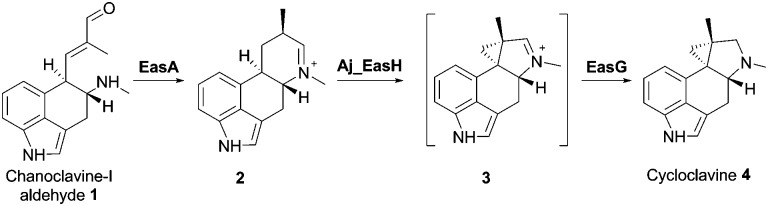
Aj_EasH catalytic reaction to generate cycloclavine **4**.

In this process, Aj_EasH is responsible for conversion of a 6-membered ring of the product of EasA to the fused 5-3 ring system observed in the cycloclavine molecule **4** (Fig. 1). The reaction catalyzed by Aj_EasH represents a highly unusual enzymatic means to construct a cyclopropyl group. The formation of a cyclopropyl moiety from a 6-membered ring by an oxidative mechanism is, to the best of our knowledge, unprecedented. Given the interest in this biochemically unique reaction, we crystallized EasH to better understand its structure and mechanism.

The initial characterization of Aj_EasH was performed using protein derived from a yeast heterologous expression system.^2^ To obtain quantities of purified enzyme sufficient for crystallization efforts, a procedure for expressing Aj_EasH in *E. coli* was developed. *Aj_easH* was initially cloned into the *E. coli* expression vector pOPIN-F, with an N-terminal His_6_-tag. However, purification using a nickel affinity chromatography resulted in inactive enzyme. Indeed, the structure of AsqJ, a close homolog of EasH, has been recently determined with nickel at the active site suggesting that the catalytic iron is readily displaced.^13^ Moreover, Aj_EasH was also inhibited by a variety of divalent metal cations such as Ni^2+^, Zn^2+^, Co^2+^ and Cu^2+^ (Fig. SI-2.5, ESI†). Therefore, *Aj_easH* was cloned into the pOPIN-J vector with a GST-tag,^14^ which enabled purification *via* glutathione affinity chromatography.

After cleavage of the GST-tag, enzyme activity was retained. The protein was further purified by size-exclusion chromatography (SI-1.2 and Fig. SI-2.3, ESI†). Subsequently, EasH was crystallized in 0.01 M ZnCl_2_ 0.1 M HEPES pH 7.0, 20% (w/v) PEG 6000 and 1 mM aKG. The crystals were cryoprotected in the same buffer with the addition of 20% (v/v) ethylene glycol prior to X-ray data collection. The structure was determined by molecular replacement to a resolution of 2.2 Å (*R*_free_ = 0.24; *R*_work_ = 0.22; PDB ID: ; 5M0T, Fig. 2a), by using the coordinates of Cp_EasH (PDB ID: ; 4NAO), a homologue from *Claviceps purpurea* that catalyzes a hydroxylation reaction (Fig. SI-2.1a, ESI†), as a starting model.^15^ In addition to Cp_EasH (40.3% sequence identity, 1.08 Å RMSD^16,17^), Aj_EasH was compared with AsqJ (PDB ID: ; 5DAP, 35.5% sequence identity, 0.82 Å RMSD^16,17^), an enzyme from *Aspergillus nidulans*, which performs two stepwise oxidations followed by non-enzymatic rearrangement on the alkaloid 4′-methoxycyclopeptin (Fig. SI-2.1b, SI-2.2 and SI-2.7, ESI†).^13,18^ Inspection of the electron density map of Aj_EasH showed clear density for aKG and iron (Fig. 2b). Numerous efforts to soak or co-crystallize Aj_EasH with substrate (chanoclavine-I aldehyde **1**) or product (cycloclavine **4**) did not result in the observation of a ternary complex. However, the location of the active site could be clearly inferred from the location of the iron and aKG cofactors. The aKG appeared to be coordinated to the iron as would be expected for a catalytically competent orientation (Fig. 2b).

**Fig. 2:**
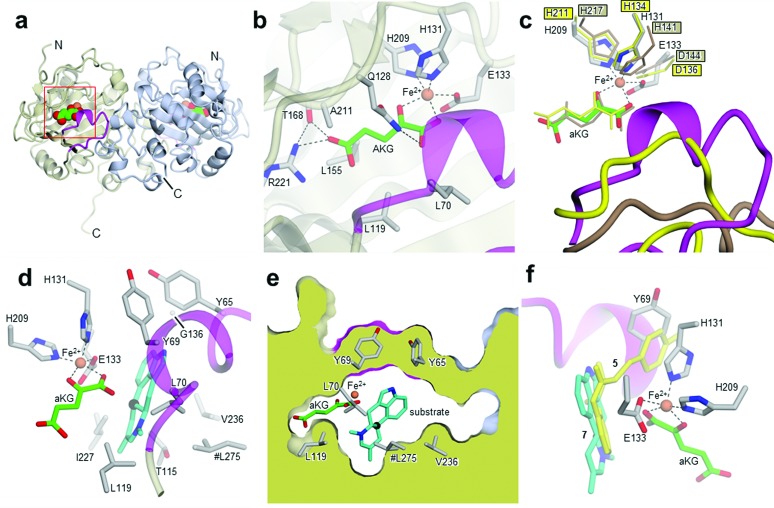
Crystal structures of Aj_EasH and its homologs. (a) Overall structure of the biological dimer of Aj_EasH (subunits in tan and blue) with magenta to highlight the lid; aKG molecules are shown in spheres with green carbons; the orange spheres represent iron. The C-terminal end of chain A adopts an extended conformation which is stabilized by crystal contacts. (b) Active site of Aj_EasH. Residues in close proximity to the iron and aKG are shown in sticks. (c) Alignment of active sites of Aj_EasH, Cp_EasH and AsqJ. Aj_EasH is colored as for part b; AsqJ is entirely in yellow; Cp_EasH is entirely in brown. The protein backbone corresponding to the lid of Aj_EasH is shown for each structure. (d) Structure of the substrate (enamine **5**; cyan carbons) manually docked into the active site of Aj_EasH. Also shown are the hydrophobic residues in the binding pocket (only the Cα atom of G136 is shown). With the exception of L275 (emphasized by the hash symbol), all the residues shown belong to the same subunit of the homodimer. C10 of the substrate lies close to the iron. (e) Cross-section through a molecular surface centered on the active site, illustrating the complete burial of the substrate binding pocket containing the docked substrate. The interior of the protein is shown in lime and the external surfaces are colored as for Fig. 2a. (f) Superposition of AsqJ/ligand, 4′-methoxycyclopeptin, (5DAQ; yellow) onto EasH/docked enamine **5** (cyan carbons). The anthranilic acid moiety of 4′-methoxycyclopeptin overlaps well with enamine **5**, but the Tyr(OMe) moiety of AsqJ substrate clashes with Y69 in the lid. Therefore Aj_EasH would not be able to accommodate 4′-methoxycyclopeptin in its current conformation.

Cp_EasH and AsqJ were initially superposed onto Aj_EasH using the Secondary Structure Matching algorithm (Fig. SI-2.2, ESI†) based on a single subunit. Then they were manually translated such that the metal ions overlapped exactly (note that in the AsqJ structure, the metal ion is nickel). This demonstrated that in the active site of Aj_EasH the positions of Fe and aKG are largely unchanged compared to Cp_EasH and AsqJ (Fig. 2c). The iron in Aj_EasH is coordinated by H131, E133, H209, in a similar fashion to AsqJ (H134, D136, H211) and Cp_EasH (H141, D143, H217). The most significant differences between these three protein structures occur in a surface loop. Within this loop there is a short helical segment (residues 52–72), which we refer to as the “lid” (Fig. 2c). This is also the most flexible region of Aj_EasH based on main-chain *B*-factors (Fig. SI-2.6, ESI†). The lid fully occludes the active site of Aj_EasH (Fig. 2b). By contrast, in all five deposited structures of AsqJ, this loop adopts essentially the same conformation, lying adjacent to a fully open active site. In Cp_EasH the loop is somewhat further away from the active site cleft and partially disordered (Fig. 2c and Fig. SI-2.7, ESI†). Clearly in Aj_EasH, the lid must undergo conformational changes to allow substrate and product interchange; whether it has specific roles in substrate capture and positioning, or in providing the necessary environment for catalysis is presently unknown.

We then examined the active site of Aj_EasH for catalytically important residues. In Aj_EasH, in addition to the residues that coordinate the iron – which have been extensively studied^19^ and were not revisited in this report – several other polar or ionizable residues are close to the active site: Y65, Y69, part of the lid (residues 64–69), D135, and Q128. We mutated these residues to alanines to explore their effects on catalytic activity (Fig. 3a and b). Quantitative kinetic constants for EasH could not be obtained in this three enzyme reaction,^2^ so only qualitative conclusions could be drawn about these mutants. However, in all cases, the variant proteins largely retained activity comparable to the wild-type protein. Overall, these results fail to identify a residue that plays a crucial role in the activity of Aj_EasH or any residue that could participate in acid–base catalysis.

**Fig. 3:**
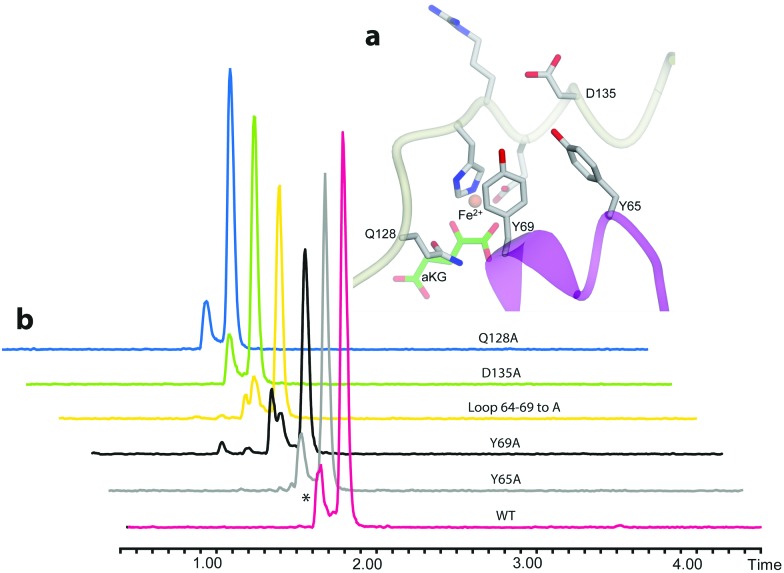
Mutational analysis of Aj_EasH. Chromatograms show MRM at *m*/*z* 239, where the major peak is cycloclavine **4**. The * represents an unidentified side product with *m*/*z* 239. Assays were performed as described in SI-1.3 (ESI†) with EasA, EasH and EasG and chanoclavine aldehyde I **1**. (a) Mutated residues in the Aj_EasH active site. The residues in the Aj_EasH active site (WT = wild type enzyme). The view is similar to that shown in Fig. 2. (b) LC-MS chromatograms presenting activity of Aj_EasH mutants.

In the absence of a crystal structure with either the substrate or the product bound to the enzyme, we docked the proposed substrate into the active site to better understand the mechanism of this transformation (Fig. 2d–f). Chanoclavine-I aldehyde **1** is the initial substrate of the reaction cascade, which is converted by the flavoenzyme EasA to form the iminium species **2**. We proposed that the enamine tautomer of **2**, compound **5**, is the actual substrate for Aj_EasH.^2^ Our X-ray structure reveals that the likely substrate binding pocket of Aj_EasH is spacious and largely hydrophobic (the pocket is delimited by Tyr65, Tyr69, Val236, Thr115, Leu119, Leu275, Leu70, Ile227 and Gly136) and, based on the relatively high B factors for the lid (residues 52–72), most probably highly plastic (Fig. SI-2.6, ESI†). Taken together with the hydrophobic nature of the substrate, this made predicting the structure of the complex difficult. Indeed, docking attempts with Autodock^20^ (SI-1.8, ESI†) led to a variety of different poses that were unlikely to correspond to catalytically competent binding modes. Nevertheless, we were able to manually place the substrate so that it did not clash with neighbouring atoms and, importantly, it was oriented such that the C10 hydrogen atom was directed towards and within 5.5 Å of the iron (Fig. 2d).

In our initial report of EasH, we proposed several possible catalytic mechanisms.^2^ Since the aKG dependent enzymes are known to catalyze hydroxylation^15^ and halogenation^21^ reactions, one potential mechanism invokes a hydroxylation or halogenation at carbon 10. The resulting hydroxyl or halide moiety could then act as a leaving group during the rearrangement to the 5-3 ring system (Fig. 4). However, given the hydrophobic active site environment and the lack of an acidic residue that could act to protonate any leaving group (Cl or OH) at carbon 10, an alternative mechanism of direct abstraction of hydrogen at C10 by the iron–oxo species followed by rearrangement should also be considered. This reaction would, in principle, not require acid–base catalysis by an active site residue (Fig. 4).

**Fig. 4:**
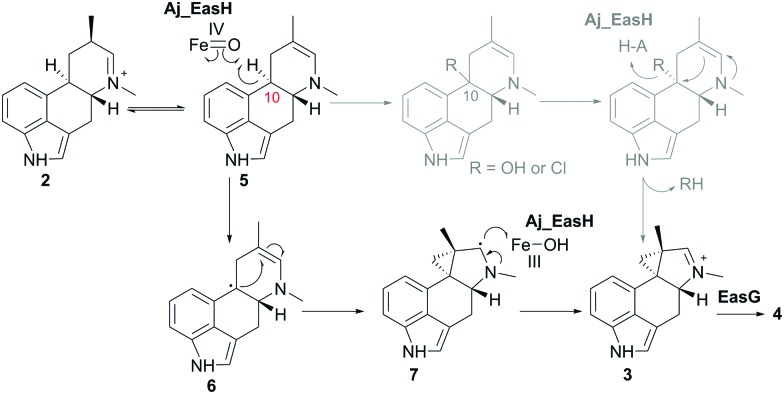
Proposed mechanism of Aj_EasH catalysis *via* radical cyclization. Alternative scenario of hydroxylation or halogenation is shown in grey.

This type of reaction must occur in a hydrophobic environment that is shielded from bulk solvent to prevent premature quenching of the radical. In addition to providing this hydrophobic environment, the active site also orients the substrate in the correct position relative to the iron cofactor. The structure of the enzyme active site must also help drive the rearrangement of the 6-membered ring to the 5-3 ring system of cycloclavine. The mobile lid (residues 52–72) likely allows conformational changes in the active site as the substrate rearranges into the product. The shape of the active site also likely controls the stereochemical course of the reaction, leading to the observed stereochemistry of the cyclopropyl group in the cycloclavine product **4**. However, the large size of the active site and the highly mobile lid make it difficult to use the contours of the active site to predict how this stereochemical outcome is achieved. The secondary radical of the putative intermediate **7** that is formed after rearrangement to the 5-3 ring system is stabilized by the presence of the lone pair of the adjacent amine. Finally, release of the positively charged iminium product **3**, which serves as the substrate for EasG, is most likely accelerated by repulsion from the hydrophobic active site cavity.

Aj_EasH catalyzes a highly unusual ring rearrangement to form the 5-3 ring system of the ergot alkaloid cycloclavine **4**. The crystal structure of this enzyme provides insight into the formation of this natural product with this rare ring framework. A neutral, radical reaction intermediate is most consistent with the structure of the active site provided by this crystallographic study. Although a ternary complex with substrate or product could not be determined, the size of the active site can clearly accommodate the substrate and product, and the highly mobile loop region that forms a lid over the active site may contribute to the overall shape of the active site, which in turn could facilitate the rearrangement of 6 to 5-3 membered ring system. Cyclopropyl group biosynthesis has been reported to occur *via* either carbocationic or carbanionic intermediates in the terpenoid family of natural product biosynthesis.^22^ To the best of our knowledge, this is the only example of oxidative radical biosynthesis of cyclopropyl group known to date.

Overall, the structure of Aj_EasH reported here supports a unique oxidative cyclization reaction which confirms the exceptional catalytic versatility of the aKG-dependent oxidases.

We would like to acknowledge Biotechnology and Biological Sciences Research Council (BBSRC) for funding this project (BB/J018171/1). For the X-ray data collection we acknowledge Diamond Light Source for access to beamline I02 under proposal MX9475, with support from the European Community's Seventh Framework Program (FP7/2007–2013) under Grant Agreement 283570 (BioStruct-X). The EasH sequence is deposited under code JE956656.
