# Supplementary material for: Structural characterization of EasH (Aspergillus japonicus) – an oxidase involved in cycloclavine biosynthesis
Source: Chem Commun (Camb). 2016 Nov 22;52(99):14306–9. doi: 10.1039/c6cc08438a (PMC5317212; doi:10.1039/c6cc08438a)
Supplement: Supplementary file 1 [file CC-052-C6CC08438A-s001.pdf]

Electronic Supplementary Material (ESI) for ChemComm.
This journal is © The Royal Society of Chemistry 2016

Supporting information

Structural characterization of EasH (*Aspergillus japonicus*) – an oxidase involved in cycloclavine biosynthesis

Dorota Jakubczyk, Lorenzo Caputi, Clare E. M. Stevenson, David M. Lawson and Sarah E. O'Connor*

Table of contents

Contents

Table of contents 1
SI-1 Supporting methods 2
SI-1.1 General materials and methods 2
SI-1.2 Cloning, overexpression, purification of Aj_EasH 2
SI-1.3 *In vitro* assays for the wild type Aj_EasH and all of the Aj_EasH mutants 2
SI-1.4 Mass spectrometry (*in vitro* assays) 2
SI-1.5 Mutagenesis 3
SI-1.6 Crystallization and structure determination 3
SI-1.7 Crystallographic methods 3
SI-1.8 Docking parameters 4
SI-2 Supporting figures 5
SI-2.1 Reactions catalysed by Aj_EasH homologues. 5
SI-2.2 Sequence alignment of Aj_EasH, Cp_EasH and AsqJ 6
SI-2.3 SDS-PAGE analysis of the Aj_EasH fractions collected during gel filtration chromatography. 7
SI-2.4 Co-elution of cycloclavine authentic standard with an Aj_EasH assay product. 7
SI-2.5 Inhibition of EasH by divalent metal cations. 8
SI-2.6 B-factors of the "lid" region (residues 52-72) 8
SI-2.7 Comparison of monomers of Aj_EasH homologues 9
SI-3 Supporting tables 10
Table S1. Primers used in this study 10
Table S2. Crystallography statistics 11
Supporting references 12

SI-1 Supporting methods

SI-1.1 General materials and methods

Chanoclavine-I aldehyde **1**, was prepared as previously reported.[1] Acetonitrile was of LC-MS grade and was purchased from Sigma-Aldrich (St. Louis, MO, USA). All other chemicals and solvents were of analytical grade and used as purchased. GST-tagged HRV-3C Protease was purchased from Acro Biosystems. All buffers and solutions were prepared in Milli-Q water. Primers for cloning were synthesized by Integrated DNA Technologies (USA) and sequencing of the constructs was performed by Sourcebioscience (UK). CloneAmp HiFi polymerase (Clontech Takara) was used for PCR amplification. A Varian Cary 50 Bio Scanning Spectrometer was used to acquire UV-Vis spectra.

SI-1.2 Cloning, overexpression, purification of Aj_EasH

For heterologous protein expression, *easH* was amplified from the previously published vector pEVE1914[2] and cloned into the *E. coli* expression vectors pOPINJ and pOPINF using the In-Fusion HD cloning kit (Clontech Takara)[3] by using the following primers: Fwd 5' AAGTTCTGTTTCAGGGCCCGACCATTACCGAAACTTCTAAGC, Rev 5' ATGGTCTAGAAAGCTTTAGGCCTTCTTAGCAACTTGATTAG.

Expression of Aj_EasH was carried out in 2YT medium supplemented by carbenicillin (100 µg/mL). *Escherichia coli* Bl21 (DE3) cells were grown to an OD600 of 0.5 prior to induction with IPTG (100 µM) and grown for 16 hours at 18°C prior to harvesting. Cells were re-suspended in buffer (50 mM K_2HPO_4, 100 mM NaCl, 10% (v/v) glycerol, pH = 7.0) and lysed by cell disruptor. Cellular debris was pelleted by centrifugation (23000 x g for 0.5 hour). EasH was purified by affinity chromatography on a 5 mL GSTrap HP column (GE Healthcare). The GST tag was then removed by treatment with 3C protease before further purification of the protein by gel filtration on a TRICORN Superdex 200 10/300 GL column (GE Healthcare). The gel filtration chromatography was performed on an Akta purifier (GE Healthcare) at 0.6 mL/min using 50 mM phosphate buffer pH 7.0 containing 150 mM NaCl as mobile phase. Elution was followed at 280 nm and 0.5 mL fractions were collected. The yield of EasH was estimated to be 2 mg per litre of culture. Fractions containing EasH, as demonstrated by SDS-PAGE, were collected and concentrated using Amicon 10 kDa MWCO centrifugal filters.

SI-1.3 *In vitro* assays for the wild type Aj_EasH and all of the Aj_EasH mutants

In vitro assays contained 0.5 µM Aj_EasA, 0.5 µM Aj_EasG, 5 µM chanoclavine-I aldehyde, 100 µM α-ketoglutaric acid, 100 µM L-ascorbic acid, 500 µM of $NADP^+$ and 10 µM $FeSO_4$ heptahydrate in 100 mM K_2HPO_4 buffer (pH = 7.5). Aj_EasH or its mutants were added at a concentration of 0.5 µM and the reactions were incubated at room temperature for 30 minutes. Aliquots (10 µL) were quenched by dilution in 40 µL mixture of 20 µL of 0.1% formic acid (in water) and 20 µL of methanol and analyzed by UPLC-MS/MS as described in section SI-1.4.

SI-1.4 Mass spectrometry (*in vitro* assays)

Ultraperformance liquid chromatography was performed on a Waters Acquity UPLC system consisting of a binary pump, an online vacuum degasser, an autosampler, and a column compartment. Separation of

the analytes was achieved on a Waters Acquity Ultra Performance BEH C18 column with 1.7 µm particle size, 2.1 x 50 mm, kept at 30 °C. Mobile phase A was water containing 0.1% (v/v) formic acid; mobile phase B was acetonitrile containing 0.1% (v/v) formic acid. The gradient profile was 0 min, 5% B; from 0 to 5 min, linear gradient to 30% B; from 5 to 7 min, back to initial conditions of 5% B. Flow rate was 0.6 mL/min and the injection volume of both the standards and the samples was 2 µL. After each injection, the needle was rinsed with 600 µL of weak wash solution (water/acetonitrile, 90:10) and 200 µL of strong wash solution (acetonitrile/water, 90:10). Samples were kept at 10 °C during the analysis.
Mass spectrometry detection was performed on a Waters Xevo TQ-S instrument equipped with an electrospray (ESI) source operated in positive mode. Capillary voltage was 3.8 kV; the source was kept at 150 °C; desolvation temperature was 200 °C; cone gas flow, 150 L/h; and desolvation gas flow, 800 L/h. Unit resolution was applied to each quadrupole. For qualitative studies, the acquisition was performed in full scan and SIR (single ion recording), while for cycloclavine **4** quantification a MRM (multiple reaction monitoring) method was used. Based on collision induced dissociation studies the first quadrupole was set on the parent ion $[M + H]^+ = 239.2$ whilst the third quadrupole was set to m/z 144.1 (cone voltage 36 V; collision energy 24 V).

SI-1.5 Mutagenesis

Mutants were generated by overlap extension PCR. The primers used to generate the mutants are reported in Supplementary Table 1. Full-length PCR products were gel purified, ligated into pPOINF andpOPINJ expression vectors and transformed into competent *E. coli* Stellar strain cells (Clontech Takara).[3] Mutant constructs were sequenced to verify the mutant gene sequence and correct insertion.

SI-1.6 Crystallization and structure determination

For crystallization, the protein was concentrated to 6.6 mg/mL and filtered through 0.1 µm PVDF filter (Durapore). α-Keto-glutarate was added to the protein solution to a concentration of 1 mM before setting up crystallization trials. PACT Premier (Molecular Dimensions) and PEGs suite (Qiagen) crystallization screens were conducted by sitting-drop vapour diffusion in MRC 96-well crystallization plates (Molecular Dimensions) with a mixture of 0.3 µL well solution and 0.3 µL protein solution. Solutions were dispensed by an OryxNano robot (Douglas Instruments). The initial screens provided several hits after four weeks incubation at 20°C. The best crystals for the complex Aj_EasH/α-keto-glutarate were obtained from condition C12 from the PACT Premier (Molecular Dimensions): 0.01 M $ZnCl_2$ buffer; pH 7.0 and 20% (w/v) PEG 6000. Crystals were harvested from the mother liquor using LithoLoops (Molecular Dimensions) and cryoprotected with well solution supplemented with 20% (v/v) ethylene glycol.

SI-1.7 Crystallographic methods

X-ray diffraction images were recorded using a Pilatus 6M detector (Dectris) on beamline I02 at the Diamond Light Source (Oxfordshire, UK), at a wavelength of 0.9795 Å, from a single crystal maintained at 100 K by a Cryojet cryocooler (Oxford Instruments). The resultant data were integrated using XDS[4] and scaled and merged using AIMLESS.[5] A summary of the data collection and processing statistics is presented in Supplementary Table 2. An initial model of the EasH/α-ketoglutarate complex was obtained by molecular replacement using PHASER[6] with the structure of Cp_EasH (PDB code 4NAO) as the search model. Two copies of the subunit were located in the asymmetric unit, which formed a

biologically relevant homodimer giving an estimated solvent content of 47%. The model was refined with REFMAC5[7] and manual corrections were made in COOT.[8] As well as the iron and αKG present in each copy of the active site, there were four zinc ions bound at crystal contacts. Additional electron density at one of these contacts was interpreted as a third molecule of αKG. The statistics of the final model are shown in Supplementary Table 2; this was deposited in the Protein Data Bank with accession code 5M0T.

SI-1.8 Docking parameters

Enamine **5** was docked into the Aj_EasH crystal structure using Autodock 4.2.[9]
The ligand (enamine) was prepared without any active torsions. The molecule was treated as rigid and the receptor consisted of the desolvated high-resolution crystal structure. The search space was defined by a 61x61x61 Å box with a 0.375 Å grid spacing and encompassed the entire active site cavity. Searches were performed using the Lamarckian Genetic Algorithm, consisting of 100 runs with a population size of 150 and 2,500,000 energy evaluations. A total of 4,574 generations were analysed and clustered with an RMS tolerance of 2 Å per cluster. This resulted in four distinct clusters, which constituted 70%, 9%, 15% and 6% of the resultant conformations, respectively.[9]

SI-2 Supporting figures

SI-2.1 Reactions catalysed by Aj_EasH homologues.

a) Hydroxylation of a peptide substrate by Cp_EasH. **b)** Two step oxidations of 4'-methoxycyclopeptin catalyzed by AsqJ.

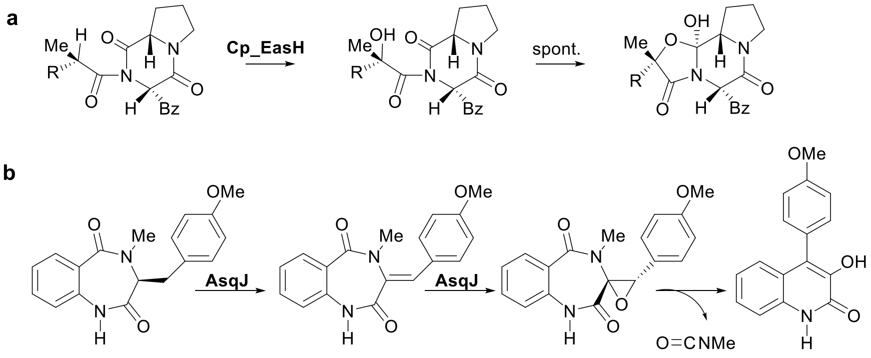

SI-2.2 Sequence alignment of Aj_EasH, Cp_EasH and AsqJ

Secondary structure elements of the Aj_EasH and AsqJ crystal structures are displayed (top and the bottom line). Numbering corresponds to Aj_EasH residues. Identical and similar amino acids are highlighted in red and yellow, respectively. Aj_EasH iron-binding amino acids (H131, E133 and H209) are indicated by blue dots, and AsqJ active site amino acids (H134, D136 and H211) are indicated by green dots. The "lid" region (residues 52-72) is underlined by a blue line. Alignment was generated using ClustalW.[10] The similarity coloring scheme used is based on physico-chemical properties. Secondary structure depiction was added using ESPript 3.[11]

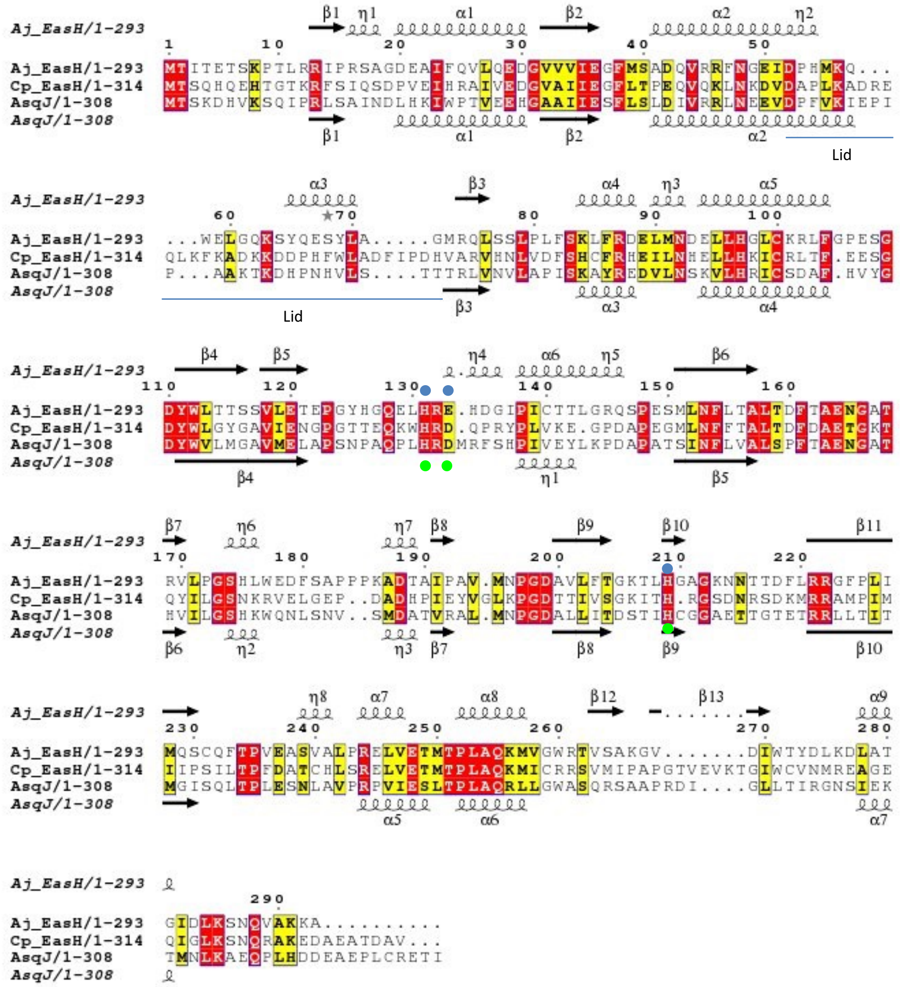

SI-2.3 SDS-PAGE analysis of the Aj_EasH fractions collected during gel filtration chromatography.

The expected size of EasH is 32.5 kDa after removal of the GST-tag. (M) Run Blue Prestained markers (Expedeon); (1-9) Protein fractions collected during gel filtration chromatography.

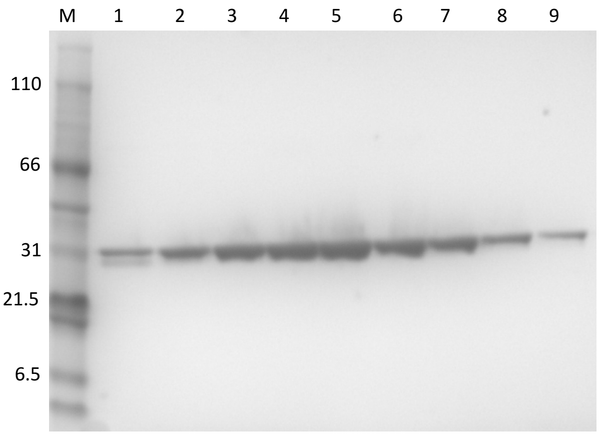

SI-2.4 Co-elution of cycloclavine authentic standard with an Aj_EasH assay product.

The figure shows UPLC/MS traces at selected ion monitoring of m/z 239 (mass of the expected enzymatic product, cycloclavine **4**) of **a)** cycloclavine **4** authentic standard and **b)** a representative *in vitro* assay product. The assays were perfomed with substrate, chanoclavine aldehyde I **1**, EasA, EasH and EasG and cofactors ($FeSO_4$, L-ascorbic acid, $NADP^+$ and aKG) as described in section SI-1.3.

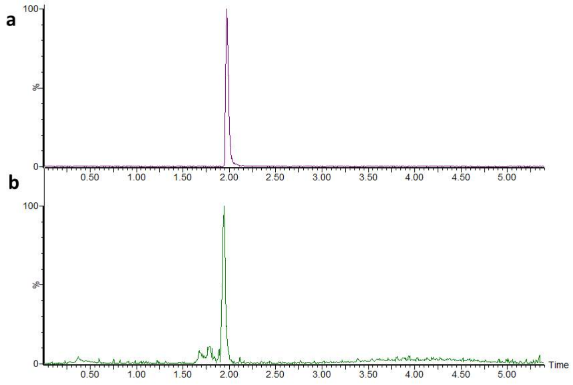

SI-2.5 Inhibition of EasH by divalent metal cations.

The figure shows UPLC/MS traces at selected ion monitoring of m/z 239 (mass of the expected enzymatic product, cycloclavine **4**) of *in vitro* assays in which different divalent cations were added to the reaction. The assays were perfomed with substrate, chanoclavine aldehyde I, EasA, EasH and EasG and cofactors ($FeSO_4$, L-ascorbic acid, $NADP^+$ and aKG) as described in section SI-1.3, except that various divalent metals were added at the start of the enzymatic reaction, as described below. a) Wild type enzyme (positive control) with no additional divalent metal added; b) Addition of 1 mM of $NiCl_2$; c) Addition of 1 mM of $Zn(CH_3CO_2)_2$; d) Addition of 1 mM of $CoCl_2$, e) Addition of 1 mM of $CuSO_4$. Inhibition of cycloclavine **4** formation by divalent metal cations has been observed in entries **b-e**.

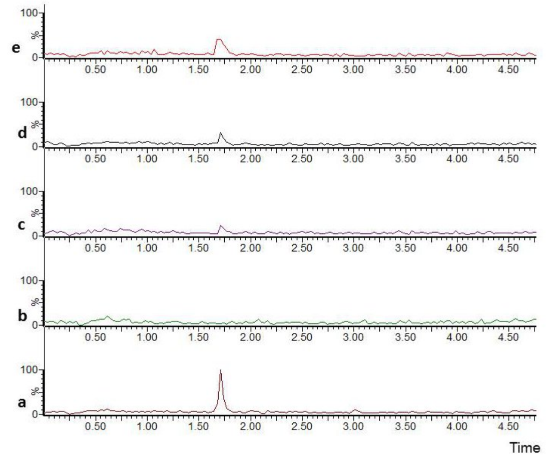

SI-2.6 B-factors of the "lid" region (residues 52-72)

Main-chain B-factors are averaged for each residue of the final model and plotted against residue number. The traces for the two subunits in the asymmetric unit are overlaid clearly illustrating that the lid regions are the most mobile in both cases.

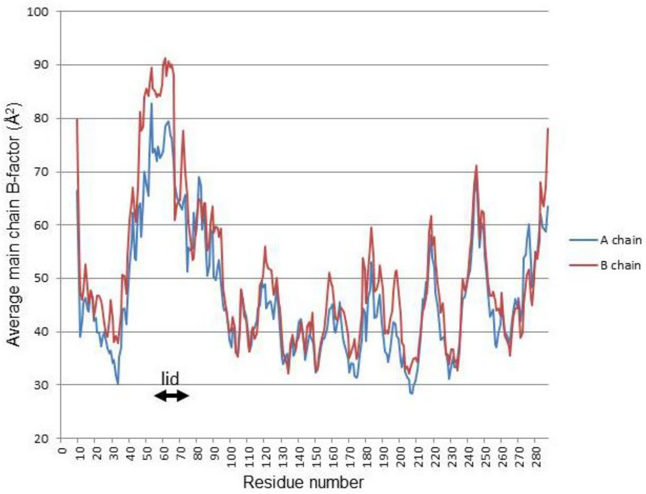

SI-2.7 Comparison of monomers of Aj_EasH homologues

The main difference is in the loop region (magenta). aKG is represented as spheres (carbon atoms are shown in green and oxygen atoms in red; the iron is shown in orange).

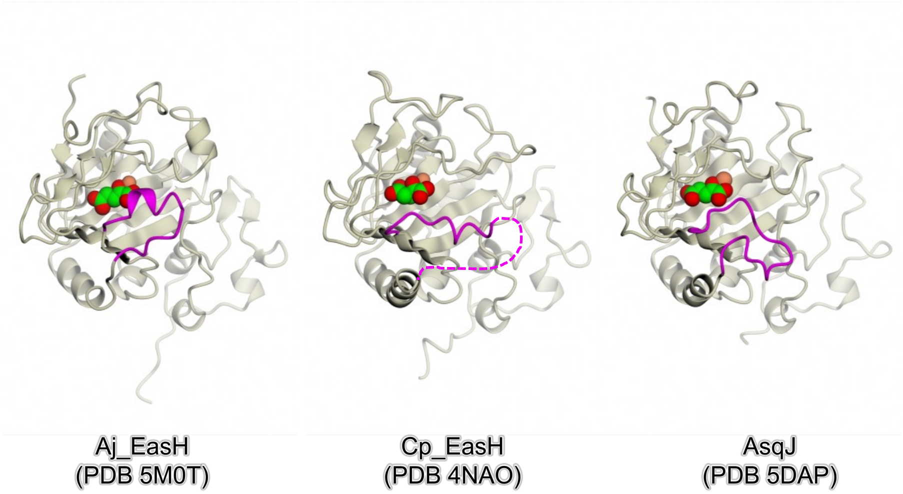

SI-3 Supporting tables

Table S1. Primers used in this study

Mutant	Forward primer	Reverse primer
Y65A	CATGAAGCAATGGGAATTGGGTCAAAAATCCGCCCAAGAATCTTACTTGGC	TGACCCAATTCCCATTGCTTCATG
Y69A	CATGAAGCAATGGGAATTGGGTCAAAAATCCTACCAAGAATCTGCCTTGGCTGGTATGAG	TGACCCAATTCCCATTGCTTCATG
Part of the lid: 64-69 to A	CATGAAGCAATGGGAATTGGGTCAAAAAGATGACCCACATTTTTGGTTGGCTGGTATGAG	TGACCCAATTCCCATTGCTTCATG
E133A	CTGAACCAGGTTACCATGGTCAAGAATTGCATAGAGCACATGATGGTATTCCAATCTG	CAATTCTTGACCATGGTAACCTGGTTCAG
D135A	CTGAACCAGGTTACCATGGTCAAGAATTGCATAGAGAACATGCTGGTATTCCAATCTG	CAATTCTTGACCATGGTAACCTGGTTCAG
Q128A	CTTCTTCTGTTTTGGAAACTGAACCAGGTTACCATGGTGCAGAATTGCATAGAGAACATG	CCTGGTTCAGTTTCCAAAACAGAAGAAG
131-133 HRE to HQA	CAGGTTACCATGGTCAAGAATTGCATCAAGCACATGATGGTATTCCAATCTGTACT	CAATTCTTGACCATGGTAACC
131-133 HRE to HRA	CAGGTTACCATGGTCAAGAATTGCATAGAGCACATGATGGTATTCCAATCTGTACT	CAATTCTTGACCATGGTAACC

Table S2. Crystallography statistics

Data collection	
Beamline	I02, Diamond Light Source
Wavelength (Å)	0.9795
Detector	Pilatus 6M
Resolution range (Å)	47.47 – 2.20 (2.26 – 2.20)
Space Group	$P2_12_12_1$
Cell parameters (Å)	*a* = 40.25, *b* = 100.17, *c* = 148.88
Total no. of measured intensities	185,902 (13,000)
Unique reflections	31,397 (2,300)
Multiplicity	5.9 (5.7)
Mean $I/\sigma(I)$	8.7 (1.3)
Completeness (%)	99.7 (99.5)
R_{merge}[a]	0.151 (1.413)
R_{meas}[b]	0.166 (1.558)
$CC_{½}$[c]	0.996 (0.539)
Wilson *B* value ($Å^2$)	34.6
Refinement	
Resolution range (Å)	47.47 – 2.20 (2.26 – 2.20)
Reflections: working/free[d]	29,876/1,521
R_{work}/R_{free}[e]	0.218/0.244 (0.357/0.364)
Ramachandran plot: favoured/allowed/disallowed[f] (%)	97.4/2.6/0.0
R.m.s. bond distance deviation (Å)	0.009
R.m.s. bond angle deviation (°)	1.38
No. of protein residues: chain A/chain B (ranges)	287 (7-293)/279 (9-287)
No. of AKG/Fe^{2+}/Zn^{2+}/water molecules	3/2/4/107
Mean *B* factors: protein/AKG/ Fe^{2+}/Zn^{2+}/water/overall ($Å^2$)	51/52/33/61/40/51
PDB accession code	5M0T

Values in parentheses are for the outer resolution shell.
[a] $R_{\text{merge}} = \sum_{hkl} \sum_i |I_i(hkl) - \langle I(hkl) \rangle| / \sum_{hkl} \sum_i I_i(hkl)$.
[b] $R_{\text{meas}} = \sum_{hkl} [N/(N-1)]^{1/2} \times \sum_i |I_i(hkl) - \langle I(hkl) \rangle| / \sum_{hkl} \sum_i I_i(hkl)$, where $I_i(hkl)$ is the ith observation of reflection *hkl*, $\langle I(hkl) \rangle$ is the weighted average intensity for all observations *i* of reflection *hkl* and *N* is the number of observations of reflection *hkl*.
[c] $CC_{½}$ is the correlation coefficient between symmetry equivalent intensities from random halves of the data set.
[d] The data set was split into "working" and "free" sets consisting of 95 and 5% of the data respectively. The free set was not used for refinement.
[e] The R-factors R_{work} and R_{free} are calculated as follows: $R = \sum(| F_{\text{obs}} - F_{\text{calc}} |)/\sum| F_{\text{obs}} |$, where F_{obs} and F_{calc} are the observed and calculated structure factor amplitudes, respectively.
[f] As calculated using MolProbity.[12]

Supporting references

1. J. Z. Cheng, C. M. Coyle, D. G. Panaccione and S. E. O'Connor, *J. Am. Chem. Soc.*, 2010, **132**, 1776-1777.
2. D. Jakubczyk, L. Caputi, A. Hatsch, C. A. F. Nielsen, M. Diefenbacher, J. Klein, A. Molt, H. Schröder, J. Z. Cheng, M. Naesby and S. E. O'Connor, *Angew. Chem.*, 2015, **54**, 5117-5121.
3. N. S. Berrow, D. Alderton, S. Sainsbury, J. Nettleship, R. Assenberg, N. Rahman, D. I. Stuart and R. J. Owens, *Nucleic Acids Res.*, 2007, **35**, e45.
4. W. Kabsch, *Acta Crystallogr., Sect D: Biol. Crystallogr.*, 2010, **66**, 125-132.
5. P. R. Evans and G. N. Murshudov, *Acta Crystallogr., Sect D: Biol. Crystallogr.*, 2013, **69**, 1204-1214.
6. A. J. McCoy, R. W. Grosse-Kunstleve, P. D. Adams, M. D. Winn, L. C. Storoni and R. J. Read, *J. Appl. Crystallogr.*, 2007, **40**, 658-674.
7. G. N. Murshudov, A. A. Vagin and E. J. Dodson, *Acta Crystallogr., Sect D: Biol. Crystallogr.*, 1997, **53**, 240-255.
8. P. Emsley and K. Cowtan, *Acta Crystallogr., Sect D: Biol. Crystallogr.*, 2004, **60**, 2126-2132.
9. G. M. Morris, R. Huey, W. Lindstrom, M. F. Sanner, R. K. Belew, D. S. Goodsell and A. J. Olson, *J. Comput. Chem.*, 2009, **30**, 2785-2791.
10. M. A. Larkin, G. Blackshields, N. P. Brown, R. Chenna, P. A. McGettigan, H. McWilliam, F. Valentin, I. M. Wallace, A. Wilm, R. Lopez, J. D. Thompson, T. J. Gibson and D. G. Higgins, *Bioinformatics*, 2007, **23**, 2947-2948.
11. X. Robert and P. Gouet, *Nucleic Acids Res.*, 2014, **42**, W320-W324.
12. I. W. Davis, A. Leaver-Fay, V. B. Chen, J. N. Block, G. J. Kapral, X. Wang, L. W. Murray, W. B. Arendall, 3rd, J. Snoeyink, J. S. Richardson and D. C. Richardson, *Nucleic Acids Res.*, 2007, **35**, W375-383.
